# Novel Lentivirus-Based Method for Rapid Selection of Inhibitory Nanobody against PRRSV

**DOI:** 10.3390/v12020229

**Published:** 2020-02-19

**Authors:** Ze-Hui Liu, Kai-Xia Lei, Guang-Wei Han, Hui-Ling Xu, Fang He

**Affiliations:** 1 Institute of Preventive Veterinary Medicine, College of Animal Sciences of Zhejiang University, Hangzhou 310058, China; zehuiliu@zju.edu.cn (Z.-H.L.); 21717417@zju.edu.cn (K.-X.L.); 11617032@zju.edu.cn (G.-W.H.); 11917012@zju.edu.cn (H.-L.X.); 2 Zhejiang Provincial Key Laboratory of Preventive Veterinary Medicine, Hangzhou 310058, China

**Keywords:** PRRSV, antiviral strategy, lentivirus based nanobody library, nanobody selection

## Abstract

The emergence and re-emergence of porcine reproductive and respiratory syndrome virus (PRRSV) has resulted in huge economic losses for the swine industry. Current vaccines are of limited efficacy against endemic circulating PRRSV variants. New strategies against PRRSV infection are in urgent need. Here, a nanobody library in Marc-145 cells is constructed for antiviral nanobodies. Nanobody encoding sequences from two non-immunized llamas were cloned to generate a pseudotyped lentiviral library. Several candidates were selected from survival cells post-PRRSV inoculation and further characterized. Nb9 was identified with strong antiviral activity. Moreover, Nb9 exerted antiviral activity via its interaction with PRRSV viral proteins, as revealed by immunofluorescence assay and Western blot. Taken together, the novel function-based screen of the lentivirus nanobody library, instead of the conventional affinity-based screen, offers an alternative strategy for antiviral reagents against PRRSV and other pathogens.

## 1. Introduction

Porcine reproductive and respiratory syndrome (PRRS) is a highly contagious viral disease that is economically important to the pig industry worldwide [1]. The etiological agent, PRRS virus (PRRSV), belongs to the genus *Arterivirus* of the family *Arteriviridae*, within the order *Nidovirales* [2]. Current vaccines available against PRRSV cannot effectively control PRRSV infection due to the extensive antigenic variation [3,4], the antibody-dependent enhancement [5,6], and the suppression of innate immune signaling pathways (IFN-α and IFN-β) [7,8]. In addition, PRRSV can persist in natural hosts for several months without any early clinical symptoms after the initial infection, potentially resulting in rapid transmission and occurrence in the herds [9,10,11]. Therefore, it is urgent for the pig industry to develop effective antiviral strategies to combat PRRSV infection.

In the past decades, a wide range of antiviral agents against PRRSV infection have been reported, including microRNAs, antisense RNA, immunostimulatory factors, botanical extracts and antibodies [12]. Nanobodies (Nbs) are variable domains of heavy chains of antibodies (VHHs) derived from camelidae and sharks. Small size and long complementary determinations of Nbs allow them to identify antigenic epitopes inaccessible to traditional antibodies [13]. In view of these features, Nbs have shown great potential in diagnostic and therapeutic applications [14]. However, it has been hampered by the selective permeability of bioactive macromolecules, thus hindering the practical application in vivo.

For PRRSV, nanobody immune libraries based on NSP9 and NSP4 proteins have been screened and tested for antiviral activity against PRRSV [15,16,17]. Two Nbs were selected using phage display. Nanobodies that are intracellularly delivered with cell-penetrating peptide HIV TAT were shown to suppress PRRSV infection by preventing viral replicase-associated polyproteins (pp1a and pp1b) from being cleaved into functional nonstructural proteins [18]. In addition, another HIV TAT-fused nanobody was reported with improved efficiency in cell uptake and anticancer activities in vitro [19]. These findings confirm the function of cell-penetrating peptides to deliver Nbs for virus inhibition.

Antibody display libraries have become one of the mainstream technologies for therapeutic antibodies. Currently, the conventional antibody library technology includes phage display, yeast two-hybrid, yeast display, mammalian cell display, and ribosome display. Phage display is one of the most common methods for antibody selection and maturation in vitro with its simplicity and versatility [20]. For phage display, candidates are typically identified based on their affinity toward specific antigens. Therefore, further cell-based function tests are strictly required to verify and characterize the antiviral activities.

Viruses are natural carriers of genetic information. Lentiviral vectors show advantages to transduce nondividing and dividing cells with ease in manipulation, simplicity, and high efficiency [21]. Therefore, lentiviral vectors are primarily used as carriers of genetic cargo for gene delivery and stable overexpression in specific cell lines, tissues, and organs [22].

In this study, a naive camelid nanobody library is constructed and followed by lentivirus-mediated delivery of the nanobody repertoire into Marc-145 cells. Several nanobodies were identified to influence PRRSV proliferation in Marc-145 cells. Among the candidates, Nb9 could potentially inhibit PRRSV when intracellular expressed in cytoplasm. Further results showed that *E. coli* expressing Nb9 protein with a cell-penetrating peptide entered Marc-145 cells efficiently and suppressed PRRSV replication by interaction with viral proteins. This lentivirus nanobody library screen paves the way for the discovery of novel antiviral strategies.

## 2. Materials and Methods

### 2.1. Cell Lines and Viruses

PRRSV-permissive Marc-145 and HEK293T cells were obtained from American Type Culture Collection (ATCC). Cells were maintained in Dulbecco’s modified Eagle medium (DMEM, Gibco, Grand Island, NY, USA) supplemented 10% fetal bovine serum (FBS, Invitrogen, CA, USA) at 37 °C with 5% CO_2_.

PRRSV titers were measured by a microtitration assay using Marc-145 cells in 96-well plates and calculated as 50% tissue culture infective doses (TCID_50_) per milliliter according to the method of Reed and Muench.

### 2.2. Isolation of Nanobody Sequence

Nanobody fragments were isolated, as described previously [23]. In brief, about 150 mL of blood sample was collected from two 6-month-old *Camelus Bactrianus*, and PBMCs were isolated by discontinuous density gradient centrifugation on Ficoll–Paque. Total RNA was extracted using the RNeasy plus mini kit (Qiagen, Hilden, Germany). The first strand of cDNA was synthesized by EasyScript^®^ Reverse Transcriptase (Transgen, Beijing, China). Nanobody fragments were obtained by a nested PCR. The first round of amplification was performed using the primer pair (CALL01 and CALL02) (Table 1), followed by the second round using the primers XbaI-Nb-F and NotI-Nb-R. The resulting 400 bp product of Nb genes was extracted using a SanPrep Column DNA gel extraction kit (Sangon Biotech, Shanghai, China).

### 2.3. Lentivectors Production and Concentration

**Figure 1:**
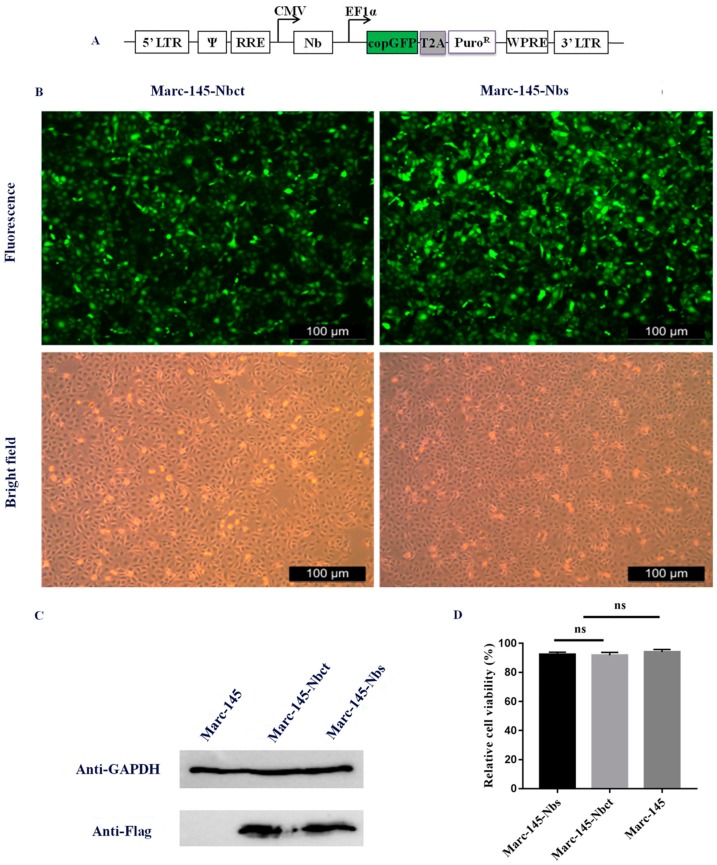
Marc-145 cells stably expressing nanobodies. (**A**) Schematic diagram of lentivirus construct for delivery of nanobodies into cells. CMV: human cytomegalovirus promoter, Nb: nanobody encoding cDNA fragments, EF1α: human elongation factor 1α promoter, copGFP: copepod green fluorescent protein (copGFP), T2A: a self-cleaving 2A peptide derived from a virus of the insect Thosea asigna, Puro^R^: puromycin-resistant marker. (**B**) Cell lines were imaged using fluorescent microscopy (magnification, 100×). (**C**) Expression of nanobody was also detected by Western blot using anti-Flag monoclonal antibody. (**D**) Cell viability assay was performed using MTT assay according to the manufacture’s instruction (ns, not significant, *p* > 0.05).

Nb encoding fragments were cloned into lentivirus vector pCD513B-1 (SBI, Mountain View, CA, USA), according to the manufacturer’s instructions. A nanobody (Nbct) without significant influence on PRRSV multiplication was used for negative control. Lentivirus expression cassette consisted of constitutive human cytomegalovirus (CMV) promoter for transcription of nanobody insert and constitutive elongation factor 1α (EF1α) promoter for transcription of copepod green fluorescent protein (copGFP) and puromycin-resistant marker (Puro^R^) (Figure 1A). CopGFP served as a reporter for the transfected/transduced cells, and Puro^R^ allowed for the selection of the transduced cells. The recombinant lentivirus vectors were co-transfected with three helper plasmids (pGag/Pol, pRev, pVSVG) into HEK293T cells using Lipofectamine™ transfection reagent (Invitrogen), as described by the manufacturer. To obtain high titer viral stocks for the subsequent experiment, the virus supernatant was harvested 48–72  h after transfection and concentrated as described previously [24]. Briefly, the virus-containing medium was overlaid on a sucrose-containing solution (20 mM Tris, pH 7.0, 150 mM NaCl, 1 mM ethylene diamine tetra acetic acid [EDTA]) at a 4:1 *v/v* ratio and centrifuged 10,000× *g* at 4 °C for 4 h. After centrifugation, the supernatant was carefully removed, and the virus pellet was resuspended in PBS buffer.

### 2.4. Construction and Selection of Stable Cell Line

First, 60% confluent Marc-145 cells were seeded in a sterile 60 mm cell culture dish. At 24 h after cultivation, cells were changed to fresh medium supplemented with 8 μg/mL polybrene for better transduction efficiency. Target Nbs expressing and control lentiviruses (MOI = 20) were inoculated for 12 h before cells were changed to selective medium. Viable cells with stable integration of the lentivirus construct in the genome were selected by puromycin (8 μg/mL) (Invitrogen). Marc-145 cells infected with Nb library lentivirus particles were designated as Marc-145–Nbs, while cells with control Nb lentivirus (named as Marc-145–Nbct) were generated as control. After transduction and selection, Marc-145–Nbs were trypsinized with 0.25% trypsin and pooled. The total number of transduced cells was calculated using Trypan blue staining assay. A small fraction of cells were subcloned and amplified for PCR analysis of Nb insertion rate. Passage 3 of Marc-145–Nbs and Marc-145–Nbct was used for an MTT assay. Western blot assay was performed to examine the overexpression of Flag-tagged Nbs, as described below.

### 2.5. Selection of PRRSV-Resistant Marc-145–Nbs

To screen Nbs with antiviral activity, Marc-145–Nbs was subject to PRRSV infection. Firstly, cells that stably express nanobodies were seeded into 6-well cell culture plates and infected at 10 MOI of PRRSV JK100 strain. Cells were incubated with viruses for 1 h and changed to fresh medium for 96–120 h. Cytopathic effect (CPE) and survival rate were monitored daily for cells with PRRSV-resistance (capable of expressing Nbs with antiviral activity); Subsequently, PRRSV-resistant cells were subcloned by limited dilution in 96-well plates. After amplification, PRRSV-resistant subclones were subjected to another round infection to exclude false positives. Consequently, individual surviving subclones from the second round PRRSV infection selection were amplified, and Nb coding DNA fragments stably integrated into the host genome were verified by PCR amplification and sequenced. PRRSV-infected cells were collected and analyzed by real-time PCR to evaluate the viral multiplication level. RT-qPCR was performed using ChamQ Universal SYBR qPCR Master Mix (Vazyme, Nanjing, China) and N-F/N-R primer pairs (Table 1), as described by the manufacturer. β-Actin mRNA served as an internal reference. The data were analyzed using the 2^−ΔΔCt^ method [25].

### 2.6. Western Blot of Marc-145–Nbs

Marc-145–Nbs and Marc-145–Nbct were collected and subject to RIPA lysis buffer (Beyotime, Shanghai, China). Extracted protein samples were separated using 12% SDS-PAGE and then transferred to PVDF membranes. The membranes were blocked with 5% (*w/v*) skimmed milk in PBST for 2 h at room temperature and incubated with anti-Flag tag monoclonal antibody at 37 °C for 1 h. After rinsing five times, blots were incubated with HRP-coupled goat anti-mouse IgG (1:5000 dilution, Sungene, Tianjin, China) at 37 °C for 1 h. After rinsing five times, the imunoreactive signals were detected by Super Signal West Pico/Femto chemiluminescent substrate (Thermo Scientific, IL, USA).

### 2.7. Nb sequence Analysis

To obtain sequences encoding antiviral Nbs, surviving Marc-145–Nbs clones were subject to genome DNA isolation, followed by PCR amplification using the primers CD513B-F and CD513B-R (Table 1). The resulting fragments of the expected size were extracted using a SanPrep Column DNA gel extraction kit (Sangon Biotech, Shanghai, China), ligated into a pClone007 blunt easy vector (TsingKe, Hangzhou, China) and sequenced. Multiple amino acid sequence alignment analyses were conducted using MegAlign software.

### 2.8. Production of Soluble Nbs

The Nb encoding sequence was derived from selected Marc-145–Nbs. The product was ligated to a modified pET-30a expression vector (pET-30a–Nbs). The correct recombinant plasmid was transformed into an *E. coli* strain, Transetta (DE3) (Transgen, Beijing, China). Recombinant protein targets were induced by IPTG with a final concretration of 1 mM at 16 °C for 12 to 16 h. Induced bacterial was resuspended in binding solution (25 mM Tris, 200 mM NaCl, 10 mM imidazole, 100 μg/mL lysozyme, pH 8.0). After ultrasonic disintegration and centrifugation, cell lysate supernatant was filtered using a 0.45 μM syringe filter and purified using ProteinIso Ni-NTA Agarose (Transgen, Beijing, China), as described by the manufacturer. Eluted protein targets were loaded onto a 3000 MW tube centrifugal dialyzer (Merck Millipore, Billerica, MA, USA) for concentration and buffer replacement for PBS. Protein samples were quantified by a BCA protein assay kit (Beyotime, China) and analyzed in SDS-PAGE and Western blotting. Sample aliquots were preserved at −80 °C until subsequent experiments. Cytotoxic effect of Nbs on Marc-145 cells was determined using MTT cell viability assay (Invitrogen, USA) according to the manufacturer’s instructions. Briefly, 1 × 10^4^ cells/well were seeded in a 96-well-plate. Different diluted Nbs (10–160 μM) or PBS was added to each well after overnight culture. After 48 h incubation, MTT solution (0.2 mg/mL) was added, incubated at 37 °C for 4 h. The supernatant was removed, and colored formazan crystal in each well was dissolved and measured using a microplate reader (BioTek). The cell survival rate was read as (OD_570_ of Nb treatment)/(OD_570_ of PBS treatment) × 100%.

### 2.9. Internalization Assays

Nbs have been reported to penetrate tissues and cells [17,26,27]. The internalization activity of Nb9 was investigated in the Marc-145 cell model using an indirect immunofluorescence assay (IFA). Monolayer cells were incubated with 80 μM of each Nb for 4 h and extensively washed to remove unbound Nbs. Subsequently, cells were collected and fixed in methanol/acetone solution and blocked with 5% skimmed milk. After being rinsed twice with PBS buffer, cell staining was performed with mouse anti-His6 mAb and FITC-coupled goat anti-mouse antibody (A-10679, Invitrogen, 1:1000 dilution). Cell nuclei were visualized with DAPI staining (1 μg/mL). After thoroughly washing, the fluorescence signal was observed using an inverted fluorescence microscope (Olympus, Corporation, Tokyo, Japan).

### 2.10. Nb9 Antiviral Assays

Marc-145 cells were pre-seeded into 6-well cell culture plates and inoculated with JK100 at an MOI of 0.01. After 1 h interaction, the virus-containing medium was replaced with fresh medium supplemented with 80 μM of each Nb. Cells were fixed in 80% acetone at 48 h post-infection (hpi), and blocked with 5% nonfat milk. Blocked cells were stained sequentially with PRRSV N-specific mouse monoclonal antibody VH13 and FITC goat anti-mouse polyclonal antibody (Invitrogen, 1:1000). Fluorescence signals were visualized by an inverted fluorescence microscope. Cultures were harvested for titration of progeny viruses as standard procedure.

### 2.11. Detection of Interaction Targets of Nb9

PRRSV-infected or mock Marc-145 cells were harvested and fixed in methanol/acetone solution at −20 °C overnight for IFA. The putative binding target located in PRRSV-infected or mock-infected Marc-145 cells was probed using His6 tagged Nb9, followed by anti-His6 rabbit pAb, and, finally, FITC goat anti-rabbit antibody (Invitrogen, 1:1000) was used. PRRSV infection in Marc-145 cells was determined using the PRRSV N-specific mouse monoclonal antibody VH13, followed by Alexa Fluor 555 conjugate donkey anti-mouse IgG (Invitrogen, 1:1500). After each step, cells were extensively rinsed with PBS to remove unbound antibody. Cell nuclei were stained with DAPI staining (1 μg/mL) for 10 min at room temperature, Signals of Nb9 or Nbct (FITC, Green), PRRSV N (Alexa Fluor 555, red) and cell nuclei (DAPI, blue) were visualized by inverted fluorescence microscope. Meantime, PRRSV-infected or mock Marc-145 cells were harvested for Western blot. The putative binding target located in PRRSV-infected or mock-infected Marc-145 cells was probed using His6 tagged Nb9, followed by anti-His6 mouse monoclonal antibody. PRRSV infection in Marc-145 cells was determined using PRRSV N-specific mouse monoclonal antibody VH13. The membrane was blocked and incubated with His6 tagged Nb9 (20 μM) at 37 °C for 1 h. After washing three times, the blots were incubated with anti-His6 mouse monoclonal antibody and PRRSV N-specific mouse monoclonal antibody VH13 at 37 °C for 1 h, and then HRP-coupled goat anti-mouse IgG (1:5000 dilution, Sungene, China) was added at 37 °C for 1 h. The immunoreactive bands were visualized as described above.

### 2.12. Statistical Analysis

Independent experiments were performed at least three times. The statistical difference was analyzed by the *t*-test when two groups were compared, or by one-way analysis of variance (ANOVA), when more than two groups were compared. The *p*-values < 0.05 (**) were considered statistically significant.

## 3. Results

### 3.1. Construction of a Lentivirus-Delivered Nanobody Library from Two Non-Immunized Camels

To construct the camel naïve Nb library, Nbs encoding cDNA fragments were obtained from PBMCs of two non-immunized camels and ligated into a lentiviral vector. Lentiviral vectors containing Nb repertoires (pCD513B-1–Nbs) consist of approximately 1.97 × 10^9^ individual colonies based on colony-forming units assay. Fifty clones were randomly selected and analyzed in colony PCR amplification. Then, 96% (48) of candidates exhibited inserts with the expected size of Nb genes. Variable Nb sequences were identified from inserts, resulting in the initial library with sufficient sequence random diversity for subsequent selection.

### 3.2. Generation of a Stable Cell Pool (Marc-145–Nbs) for Nb Expression

Nb and control lentiviruses (MOI = 10) were individually transduced to Marc-145 cells for library delivery. Positive recombinant Marc-145–Nbs and Marc-145–Nbct cells were obtained after four times of subculture and puromycin selection. The results demonstrated that Nb genes with the expected size can be overexpressed in lentiviruses-transduced cells (Figure 1). The transduced Marc-145–Nbs pool is composed of 3.5 × 10^7^ cells, around 92% of which harbored variable Nb inserts. The transduced Marc-145–Nbct cells pool was also obtained at a 96% Nbct insert rate (data not shown). Moreover, in cell proliferation assays, no significant difference was observed among Marc-145–Nbs, Marc-145–Nbct, and Marc-145 cells, indicating that nanobody proteins induce neither cytotoxicity nor any side effect on cell proliferation (Figure 1). Taken together, stable cell lines were successfully established overexpressing Nb repertoires.

### 3.3. Selection of PRRSV-Resistant Nbs by Marc-145–Nbs Based Antiviral Assays

**Figure 2:**
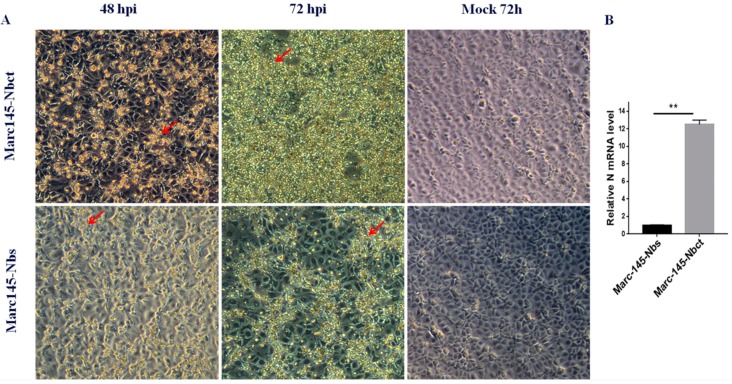
Selection of PRRSV-resistant Marc-145–Nbs. Marc-145–Nbs and Marc-145–Nbct cells were inoculated at 10 MOI of PRRSV JK100 strain. Cells were observed for cytopathic effect (CPE, indicated by red arrows) at 48 and 72 hpi, and mock-infected Marc-145–Nbs and Marc-145–Nbct cells were used as a control (magnification, 100×) (**A**). Cells were collected at 72 h post infection and relative PRRSV N mRNA levels were quantified by real-time PCR. Beta actin served as internal reference (**, *p* < 0.05) (**B**).

**Figure 3:**
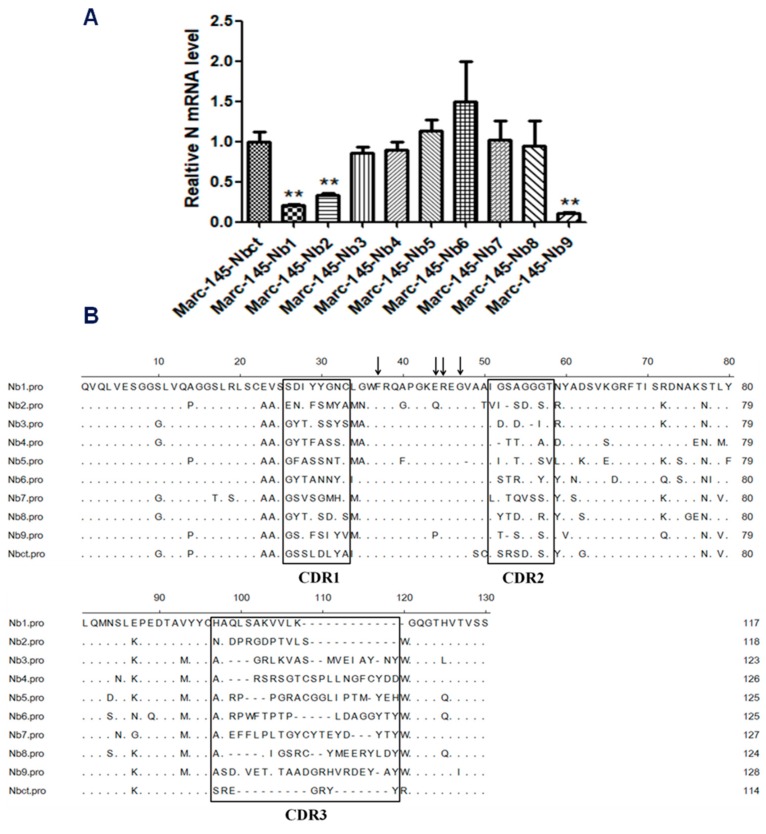
Characterization of Marc-145–Nbs subclones after PRRSV infection and sequences analysis. (**A**) Amplified clones of Marc-145–Nbs were incubated with PRRSV JK100 (1 MOI). After culturing for 48 h, cells were harvested and relative PRRSV N mRNA levels were detected using real-time PCR. Beta actin served as internal reference (**, *p* < 0.05). (**B**) Sequence alignment of selected antiviral Nbs. Complementarity-determining regions (CDRs) are denoted by the thick-line boxes. Four conservative hallmark residues of Nbs in FR2 (Val37Phe, Gly44Glu/Lys, Arg45Leu, and Trp47Gly) are denoted by the arrows.

Marc-145–Nbs or Marc-145–Nbct cells were challenged with PRRSV strain JK100 at an MOI up to 10. CPE and cell viability were monitored daily. As shown in Figure 2, PRRSV induced typical CPE in cells. It was clear that Marc-145–Nbs appeared with a slight CPE while severe CPE was observed in control cells Marc-145–Nbct. After the first selection, the viral multiplication level in cells was quantified by real-time PCR. PRRSV N mRNA levels in Marc-145–Nbs decreased significantly (Figure 2). PRRSV-resistant Marc-145 cell candidates from the first selection were subject to subclone by limited dilution in 96-well plates. Then, 285 subclones were obtained and amplified. After the second round selection, 21 Marc-145–Nbs subclones were enriched based on resistance to virus infection. It is notable that several Marc-145 subclones harbored same Nb inserts, as revealed by sequence analysis. Hence, 21 PRRSV-resistant subclones were conbined into 9 subclones. Nine antiviral Nbs were compared and characterized using sequence alignment (Figure 3). The sequence variability was observed in three CDRs of Nbs, while the rest part was well conserved. Moreover, PRRSV replication in these subclones was tested by real-time PCR. Compared to the control cell line Marc-145–Nbct, PRRSV N mRNA levels were significantly down-regulated in Marc-145–Nb1, Marc-145–Nb2, and Marc-145–Nb9 (Figure 3), indicating that Nb1, Nb2, and Nb9 developed significant antiviral activity.

### 3.4. Characteristic Analysis of Soluble Nbs in Marc-145 Cells

**Figure 4:**
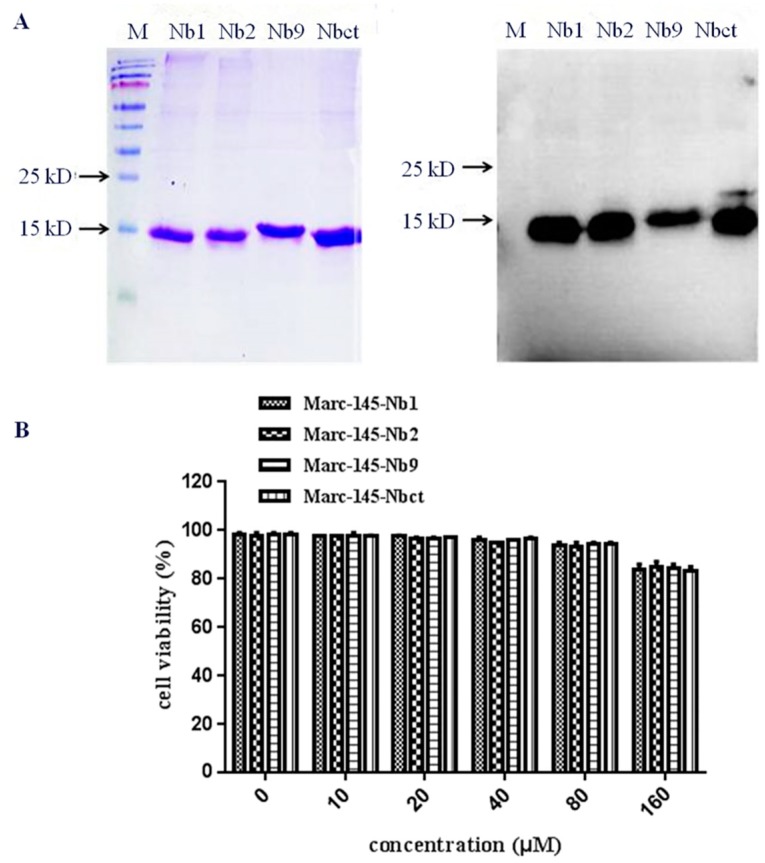
Purification and toxicity analysis of nanobodies. NLS-A cell-penetrating peptide fused His-tagged Nb proteins were purified and analyzed by SDS-PAGE and Western blot using His6 mAb (**A**). Cell toxicity of purified nanobodies was determined using MTT assay (**B**).

To verify the optimal concentration of Nbs in Marc-145, an MTT assay was performed with Nbs in Marc-145 cells. Firstly, Nb1, Nb2, Nb9, and control nanobody Nbct were expressed in *E. coli* with a 6×His tag at C terminal and an NLS-A cell-penetrating peptide [28]. Protein products of 15 kDa were purified and dialyzed (Figure 4). As shown in Figure 4, the cell viability of Marc-145 was approximately 100% at nanobody concentrations less than 80 μM. Compared with untreated control cells, cell viability was rapidly reduced to less than 85% at the nanobody concentration of 160 μM. This result revealed that Nbs was permissive to Marc-145 cells at concentrations below 160 μM.

**Figure 5:**
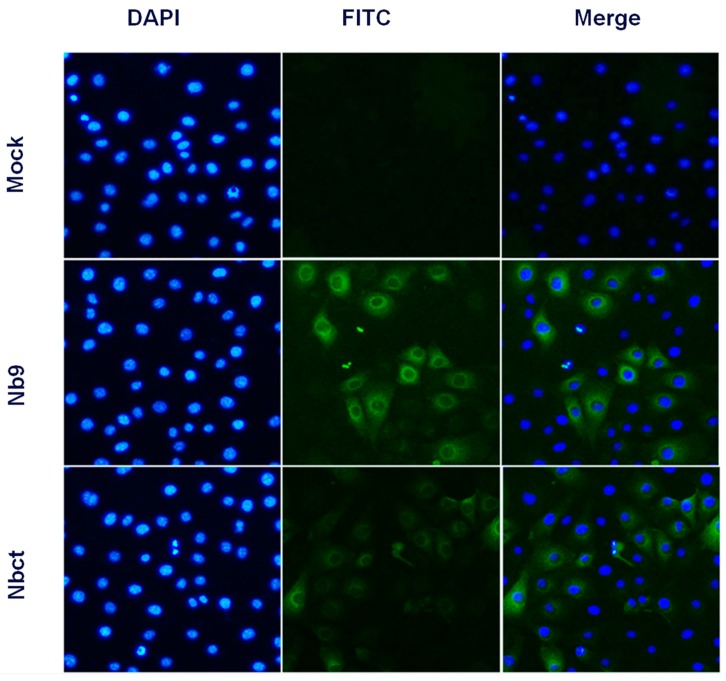
Internalization of Nb9 and Nbct in Marc-145 cells. Cells were incubated sequentially with 80 μM of each Nb, mouse anti-His6 mAb, and FITC-conjugated goat anti-mouse IgG. Signals of Nb9 or Nbct (green) and cell nuclei (blue) were visualized by inverted fluorescence microscope (magnification, 400×).

Given that intracellular expression of Nb1, Nb2, and Nb9 developed significant antiviral activity, the internalization activity of Nbs in cells was evaluated. With anti-His6 immunofluorescence, Nb9 and Nbct were clearly detected in Nb incubated Marc-145 cells, while no immunofluorescence signal was observed in untreated Marc-145 cells (Figure 5). These results indicated that Nbs delivered by NLS-A cell-penetrating peptide were successfully internalized in Marc-145 cells.

### 3.5. Antiviral Activity of Nbs Against PRRSV Infection

**Figure 6:**
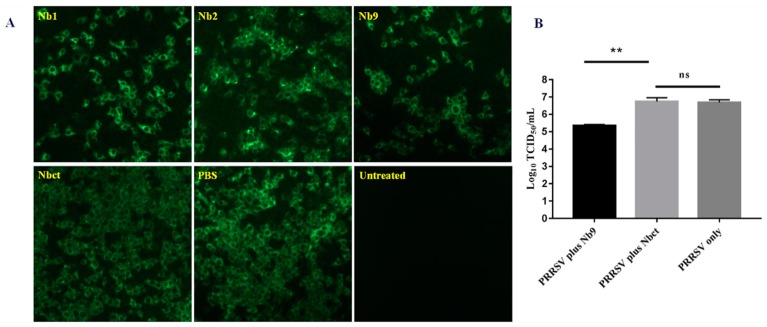
Antiviral effects of cell-penetrating peptide delivered Nb proteins on PRRSV replication. Marc-145 cells were infected with PRRSV JK100 at MOI of 0.01 for 1 h and treated with 80 μM each Nb. At 48 hpi, the cells were fixed for indirect immunofluorescence assay (IFA) using PRRSV N-specific mAb, followed by FITC-conjugated goat anti-mouse IgG (magnification, 200×) (**A**). Additionally, cultures were harvested for titration of progeny viruses (**, *p* < 0.05; ns, not significant, *p* > 0.05) (**B**).

To evaluate the effect of delivered intracellular Nb proteins on PRRSV replication, an antiviral assay was performed as mentioned above. As shown in Figure 6, significantly less PRRSV positive cells were observed with either Nb1 or Nb9 than the PBS control, while Nb2 induced a slight decrease in fluorescent cell number. The group with Nbct showed no decrease in PRRSV viral antigen expression as compared with the PBS control group. These results confirm that intracellularly delivered Nb proteins, especially Nb9, display strong anti-PRRSV activity.

**Figure 7:**
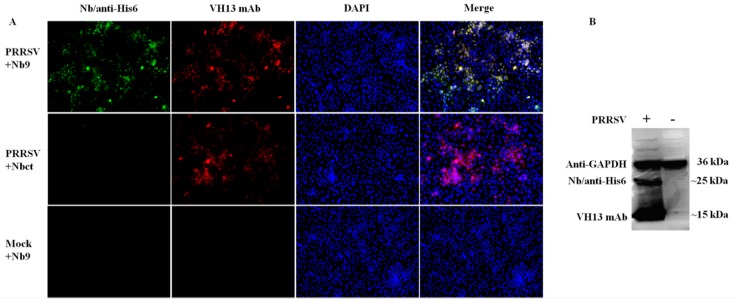
Detection of the interaction between viral proteins and Nb9. PRRSV-infected or mock-infected Marc-145 cells were collected at 36 hpi for IFA (**A**) and Western blot (**B**). (**A**) The putative binding target of Nb9 was probed using His6 tagged Nb9, then anti-His6 rabbit pAb, followed by FITC goat anti-rabbit antibody. PRRSV infection was detected using PRRSV N-specific mouse monoclonal antibody VH13, followed by Alexa Fluor 555 conjugate donkey anti-mouse IgG. Signals of Nb9 or Nbct (FITC, green), PRRSV N (Alexa Fluor 555, red) and cell nuclei (DAPI, blue) were visualized by inverted fluorescence microscope (magnification, 100×). (**B**) The putative binding target of Nb9 was detected with His6 tagged Nb9 and anti-His6 antibody. PRRSV N protein was detected with PRRSV N-specific mouse monoclonal antibody VH13. GAPDH was detected as the internal controls.

To elucidate the mechanism associated with the antiviral activity of Nb9, the potential interaction targets of Nb9 were explored in PRRSV infected Marc-145 cells. As shown in Figure 7A, in PRRSV-infected cells, Nb9 detected specific target and developed remarkable immunofluorescence (green) signal, while Nbct did not detect any signals. Besides, no signals were detected by Nb9 in mock-infected cells. Further, a Western blot confirmed these findings. It is notable that Nb9 detected a specific immunoreactive band (~25 kDa, Figure 7B). These results indicate that the antiviral activity of Nb9 is mediated by the interaction with PRRSV viral proteins, instead of cellular targets.

## 4. Discussion

PRRSV is closely related to the porcine respiratory disease complex (PRDC) syndrome that has threatened the global pig industry for several decades. Currently, there are concerns for the biosafety and efficacy of licensed vaccines, such as viral shedding, recombination with field strains, reversion to virulence, and failure to trigger a protective immune response against heterogeneous isolates [29]. In addition, experimental genetically-engineered subunit vaccines are not capable of eliciting an adequate protective immune response, even against challenges from homologous PRRSV strains [30]. Therefore, it is necessary to develop PRRSV-specific treatment for infected herds or novel antiviral strategies to broaden the knowledge of anti-PRRSV targets.

In comparison to conventional antibodies, nanobodies are characterized by low immunogenicity in primates, a small size, and easy accessibility to epitopes. Moreover, nanobodies often develop promising yields in prokaryotic and eukaryotic expression systems with high stability, making it easy to scale-up production for any therapeutic purpose. Thus, nanobodies are ideal candidates for disease diagnosis and treatment [31,32,33], including animal pathogens like PRRSV.

Nb library screens with phage display are time-consuming and costly. The candidates identified from the method are likely redundant for certain antigens [34,35]. The nanobody repertoire from immunized or non-immunized camelids is typically ligated into a vector for recombinant phage rescue, which is subsequently screened for Nbs of interest based on the affinity to immobilized antigens [35,36,37]. Several nanobodies targeting viral proteins have been isolated using phage display with antiviral activity [14,38,39]. However, it requires extra studies to determine the antiviral activities for these nanobodies derived from phage display. Moreover, many screens were conducted based on those recombinant viral targets. Most identified nanobodies bind to conformations in the given purified proteins, while other candidates against transient conformations or protein complexes are missed in these conventional approaches. These affinity-based methods sometimes cause confusing results. For example, during PRRSV infection, the immunodominant epitope A, acting as a decoy, elicits most of the antibodies specific to GP5 and delays the occurrence of neutralizing antibodies targeting epitope B by at least 21 days [40]. Therefore, affinity selection has great limitations in the selection of antiviral nanobodies.

In a selection guided with antiviral function, Nb expressing cells resist PRRSV infection, which may be caused by the interference in the virus life cycle, the inhibition in cell death, or relief of virus-induced CPE [17,41]. For the present function-based screen of the lentivirus nanobody library, PRRSV-resistant Marc-145 cells were also enriched upon lethal dose of PRRSV infection. Nine different Nbs were identified and characterized in sequence alignment (Figure 3). Moreover, limited PRRSV replication in these nine subclones indicated intracellularly expressed Nb1, Nb2, and Nb9 developed potential antiviral activity. These results indicate that this antiviral function-based selection served as an alternative strategy for antiviral reagent development.

For the development of anti-PRRSV nanobodies, a nanobody repertoire from two non-immunized llamas was ligated into a lentivirus vector to generate a pseudo-typed lentiviral Nb library. In the present study, a non-immunized nanobody repertoire was chosen, as camels are resistant to PRSSV infection. Therefore, the usage of a naïve Nb library may increase the chances of identifying novel PRRSV inhibitors. Besides, the method provides the advantages of the improved diversity of candidates and the exclusion of antibodies against known targets, which allows for the development of novel binders for any potential antigenic epitope (or pathogen) [42,43].

Nbs have been intensely investigated for diagnostic and therapeutic purposes based on beneficial biological and pharmacological properties. However, the application has been limited due to poor penetration into cells, especially for those intracellular targets. Cell-penetrating peptides (CPPs) have shown great potential in cell membrane permeability, thus being widely used for delivery of biologically active molecules, cargoes, and compounds into cells [44]. It has been reported that by fusion with CPPs, nanobodies can penetrate cells and display considerable antiviral activity [17,45]. Recently, an NLS-A CPP from PCV2 Cap was reported with efficient cellular uptake as compared to the well studied HIV TAT CPP. For antiviral agents, effective cell uptake is required to enhance the efficacy in application. NLS-A CPP has no side-effects, which is observed in HIV TAT-mediated delivery [28]. Here, anti-PRRSV nanobody candidates Nb1, Nb2, and Nb9 were overexpressed as fusion proteins with NLS-A in *E. coli*. Our results demonstrated that Nbs fused with NLS-A CPP successfully entered Marc-145 cells within only 4 h (Figure 5). Furthermore, delivered Nb1, Nb2, and Nb9 in Marc-145 cells potentially inhibited PRRSV proliferation, which confirmed the antiviral activity intracellularly expressed Nb1, Nb2, and Nb9 (Figure 6). These results verified that Nb9 delivered by NLS-A CPP presents considerable antiviral activity, leading to a promising application in therapeutics.

Nb9 was identified with anti-PRRSV activity via the interaction with PRRSV encoding proteins. Generally, antiviral agents typically exert antiviral function by binding to viral receptors, viral proteins, or other host cell factors required for viral replication [41]. For the present study, Nb9 inhibited PRRSV replication by binding to a viral protein (~25 kDa), which is probably glycoprotein 5 (GP5), of similar molecular weight. GP5 plays an important role in virus receptor interaction and is responsible for eliciting neutralizing antibodies [18,46,47]. Further study will be performed to clarify the molecular mechanisms underlying the antiviral activity of Nb9.

Furthermore, this antiviral Nb selection method can also be applied to the screening of antiviral antibodies for emerging viral diseases. As selection is based on live cells, nanobodies can target any possible factors required for the viral life cycle. Obtained antiviral Nbs targeting host restricted factors for viral uptake or nuclear import may act as broad antiviral agents [41], and also provide new perspectives into the viral replication cycle. Most importantly, identified antiviral Nbs may be combined with mass spectrometry or Nb-target complex structure analysis to identify potential neutralizing epitopes located in viral structural proteins, viral receptors, or other host factors required for viral life cycle. Meantime, this method is not limited to virus-induced CPE, but also can be conducted based on visible virus-specific signals, such as virus harboring reporter genes [48,49]. Therefore, it has great potential applicable to accelerated development of antiviral strategies, especially for these fast-evolving and public health-related viruses.

In summary, the nanobody library that was generated in this study with pseudo-typed lentiviruses offers improved sequence diversity and high quality. This Nb selection guided with antiviral function presents an innovative approach to develop antiviral agents against PRRSV and other viral pathogens, and in turn, deepens the understanding of host–pathogen interactions.

## Figures and Tables

**Table 1:** Primers used in this study.

Primer	Oligonucleotides (from 5′ to 3′)
CALL01	GTCCTGGCTGCTCTTCTACAAGG
CALL02	GGTACGTGCTGTTGAACTGTTCC
XbaI-Nb-F	GCTCTAGAATGCACCACCACCACCACCACCAGGTGCAGCTGGTGGAGTCTGGRGGAGG (R = A/G)
NotI-Nb-R	AAGGAAAAAAGCGGCCGCTTAATGGAGACGGTG ACCWGGGT (W = A/T)
CD513B-F	ATAGCGGTTTGACTCACGGGGATTT
CD513B-R	GACATCACTTTCCCAGTTTACCCCG
N-F	AGATCATCATCGCCCAACAAAAC
N-R	GACACAATTGCCGCTCACTA
β-actin-F	TCCCTGGAGAAGAGCTACGA
β-actin-R	AGCACTGTGTTGGCGTACAG
